# Early prediction of patient discharge disposition in acute neurological care using machine learning

**DOI:** 10.1186/s12913-022-08615-w

**Published:** 2022-10-25

**Authors:** Charles F. Mickle, Debzani Deb

**Affiliations:** 1 Department of Computer Science, Winston-Salem State University, Winston-Salem, NC USA; 2 Center for Applied Data Science (CADS), Winston-Salem State University, Winston-Salem, USA

**Keywords:** Machine learning, Discharge planning, Interpretability, Neurological

## Abstract

**Background:**

Acute neurological complications are some of the leading causes of death and disability in the U.S. The medical professionals that treat patients in this setting are tasked with deciding where (e.g., home or facility), how, and when to discharge these patients. It is important to be able to predict potential patient discharge outcomes as early as possible during the patient’s hospital stay and to know what factors influence the development of discharge planning. This study carried out two parallel experiments: A multi-class outcome (patient discharge targets of ‘home’, ‘nursing facility’, ‘rehab’, ‘death’) and binary class outcome (‘home’ vs. ‘non-home’). The goal of this study is to develop early predictive models for each experiment exploring which patient characteristics and clinical variables significantly influence discharge planning of patients based on the data that are available only within 24 h of their hospital admission.

**Method:**

Our methodology centers around building and training five different machine learning models followed by testing and tuning those models to find the best-suited predictor for each experiment with a dataset of 5,245 adult patients with neurological conditions taken from the eICU-CRD database.

**Results:**

The results of this study show XGBoost to be the most effective model for predicting between four common discharge outcomes of ‘home’, ‘nursing facility’, ‘rehab’, and ‘death’, with 71% average c-statistic. The XGBoost model was also the best-performer in the binary outcome experiment with a c-statistic of 76%. This article also explores the accuracy, reliability, and interpretability of the best performing models in each experiment by identifying and analyzing the features that are most impactful to the predictions.

**Conclusions:**

The acceptable accuracy and interpretability of the predictive models based on early admission data suggests that the models can be used in a suggestive context to help guide healthcare providers in efforts of planning effective and equitable discharge recommendations.

## Highlights

- The study focuses on providing early prediction of discharge locations for patients in acute care settings with severe neurological conditions based on data that are available within the 24 h of patient’s hospital admission.
- Two separate experiments were considered with separate discharge outcome classes: a binary outcome (home vs. non-home) and a multi-class outcome (home, nursing facility, rehab, death).
- Five machine learning models were developed to utilize with each experiment and provide detailed accuracy reports (confusion matrices, ROC curves, etc.).
- The study also investigates the accuracy, reliability, and interpretability of the best-performing models by identifying and analyzing the features that are most impactful to the predictions.

## Background

Acute care is typically an inpatient setting in which patients receive active care for severe injury and illness. Patients stay in the hospital for 4.8 days in the U.S for acute care on average [1]. After being discharged from the hospital, if a patient does not go to the ideal location for their continued recovery, whether it be due to the hospital’s incorrect recommendation or of their own volition, the patient could experience negative health consequences. These consequences may include falling and re-injuring themselves, resulting in a possible re-admission to the hospital [1]. Typically, medical professionals must choose from discharging the patient to their home, a rehabilitation facility, a nursing home facility, or various other potential destinations. While it is commonly known that determinants of health can impact patients in unique ways when it comes to prescribed healthcare, it is not well understood which determinants most importantly drive or influence the discharge planning from acute-care medical centers for patients under what settings. By identifying modifiable risk factors that drive up costs through hospital-acquired conditions, increased length of stay (LOS), or post-acute care needs can help providers prioritize patient care and aid in discharge planning [2].

Patients are not the only ones standing to benefit from discharge planning. Inpatient overcrowding is a significant issue for hospitals as populations increase and has been exacerbated by the 2020 pandemic fallout. Patient flow and LOS directly impact the access and quality of healthcare, as well as a hospitals financial performance [3]. In certain cases, implementing automatic discharge predictions driven by data has shown promising results to support improvements in patient flow for hospitals without adding overhead, such as extra staffing or patient capacity [3]. Despite the apparent need and benefits of this type of discharge prediction, there is a paucity of research and hard evidence of its use and effectiveness [4].

The recent proliferation of machine learning (ML) approaches has shown promising results in achieving superior prediction ability in various settings and patient conditions. The modern machine learning approaches can discover hidden patterns for non-linear high-order interactions between independent variables and yield more stable predictions than traditional statistics [5]. Currently, few tools exist that are able to automatically and accurately aid in predicting patient discharge [6]. Of those few, some have been developed with the aim of assisting in the early decision making of clinical teams rather than replacing those decisions [6]. In this context, this article explores whether various patient characteristics and clinical variables that are available within the 24 h of a patient’s hospital admission influence discharge location and the adherence to latest discharge recommendations for adults 18 years of age and older who are admitted to an acute neurological medical unit with cerebrovascular accident (CVA). This study aims to develop reliable ML models that can effectively predict discharge planning for adults with acute neurological conditions to better assist healthcare providers, as well as patients and their families, with improved care and information for determining and planning discharge recommendations.

This article considers data from 5,245 de-identified patients admitted to acute care ICU settings with severe neurological conditions and seeks to determine whether selected patient characteristics and clinical variables have enough information to potentially characterize whether the patients will be discharged to their home, a nursing facility, a rehab facility, or expire prior to discharge. The analysis of the data utilizes several patient and clinical variables including basic demographic features such as age, gender, and race; clinical features, such as height, weight, temperature; and medical risk determinations, such as Glasgow Coma Scores (GCS) and Acute Physiology and Chronic Health Evaluation (APACHE) IV [7] patient characteristics.

Focusing on the main goal to better assist healthcare providers, as well as patients and their families, with early discharge recommendations, the study carries out experiments for two different setups. The first experiment analyzes the above-mentioned dataset and considers all four discharge categories (home, a nursing facility, a rehab facility, or death) as outcomes and builds and evaluates models based on that. Based on the fact that during the early stages of hospital admission, healthcare providers and patient family members may also be interested in learning the probability of a patient discharging to ‘home’ vs. any other facilities (including funeral home arrangements in case of “death”), this study also carries out a second experiment of building and evaluating models for the binary outcomes of ‘home’ vs. ‘non-home’. This study is an extension of a previous study [8], which exclusively considered only the multi-class outcomes.

The results of this study show that the best-performing machine learning models are able to predict the discharge outcome of a patient based on their early admission data with respectable accuracies in both experiments. The accuracy, reliability, and interpretability of the best-performing model and its prediction in each experiment are also explored by using a specialized tool in identifying and analyzing the features that are most impactful to the predictions. These models, the predictions that they generate, and the interpretability that they offer could therefore be used by clinicians both in a suggestive context (prior to the clinicians making their determination on patient discharge location) or a confirmative context (after the clinicians have made their determinations) to aid healthcare providers and families in planning effective, timely, and equitable discharge recommendations for these adults with complex neurological conditions.

The rest of this article is organized as follows: "Related research" section surveys and compares the related research, "Dataset and preprocessing" section discusses the dataset and preprocessing steps, "Methodology" section elaborates the detailed methodology, "Results and discussion" section discusses the results, and "Conclusion" section concludes the article.

### Related research

A number of previous studies have also explored the use of ML in predicting patient discharge locations. Table 1 surveys and compares all previous studies, as well as this current study, concerning predictive discharge dispositions. As noted in Table 1, the focus of the presented research is to predict the discharge locations of patients in the acute neurological care setting admitted with CVA diagnoses using only patient characteristics that would be available at admission (within the first 24 h) and to analyze the significance and interpretability of the predictions, which differentiates this study with the other related studies. The determined need and motivation for this study is based on the changing healthcare needs and risk factors associated with discharge planning where an earlier prediction of expected discharge location can benefit healthcare providers as well as patients and their families.

**Table 1:** Descriptive table of ML-based predictive works on discharge disposition

Author	Setting	Discharge Outcome	Summary	Models Used
Goto et al. [9]	Asthma/COPD patients in the emergency dept	Binary (ICU vs. non-ICU hospitalization)	Compares the four models’ predictive capability to a baseline logistic regression concluding that the ML models markedly improved prediction capability	Lasso regression (LR), Randon forest (RF), Boosted decision tree (BDT), Artificial Neural network (ANN)
Karhade et al. [10]	Elective inpatient surgery for lumbar degenerative disc disorders	Binary (routine vs non-routine postoperative discharge)	Created an open-access web application for healthcare professionals that showed promising results for preoperative prediction of non-routine discharge	ANN, Support vector machine (SVM), Bayes point machine, BDT
Greenstein et al. [11]	Post-operative discharge after total joint arthroplasty (TJA)	Binary (skilled nursing facility vs. elsewhere)	Developed an EMR-integrated prediction tool to predict discharge disposition after TJA	ANN
Ogink et al. [12]	Post-operative discharge after degenerative spondylolisthesis	Binary (home vs. non-home)	Similar to [5], compares a set of predictive model’s performance after elective spinal surgery	ANN, SVM, Bayes point machine, BDT
Cho et al. [13]	Post-stroke acute care	Binary (home vs. facility)	Compares the performance of four interpretable ML models on post-stroke discharge prediction	LR, RF, AdaBoost, multi-layer perceptron
Muhlestein et al. [14]	Post-craniotomy	Binary (home vs. non-home)	Uses 26 ML algorithms to combine the best performers into ensemble model investigate the impact of race on discharge disposition	Ensemble (various)
Muhlestein et al. [15]	Post-meningioma resection	Binary (home vs. non-home)	Similar to [10], creates an ensemble model showing significantly improved accuracy compared to traditional logistic regression	Ensemble (various)
Abad et al. [16]	ICU critical care	Multi-class (home, nursing facility, rehab, death)	Investigates the impacts of APACHE IV scores on patient discharge via an array of different ML models	LR, XGBC, RF
This research study	Post-stroke acute care	Binary (home vs. non-home) and Multi-class (home, nursing facility, rehab, death)	Compares the performance of 5 ML models in both a binary and multi-class experiment and investigates the explainability of the best-performing models	RF, XGBC, KNN, SVM, LR

### Dataset and preprocessing

The dataset utilized in this study was extracted from MIT’s eICU-Collaborative Research Database (eICU-CRD). The database was derived from the Philips Healthcare eICU telehealth system to be used in applications such as ML algorithms, decision support tools, and clinical research [17]. The e-ICU-CRD is populated with patient data from a combination of many critical care units throughout the continental U.S. who were treated as part of the Philips eICU program from 2014–2015. The database is a collection of 31 different tables concerning patient data, centered around all types of critical care patients. This dataset was selected over other relevant databases, such as MIMIC-III [18], due to the fact that it contained multi-center data rather than single-center, contained more recent data, and had an overall higher quantity of available data which is typically desirable for ML. Patients in the database are connected by a unique identifier, *uniquepid*, used as a foreign key between the tables. The datafiles of these tables were browsed to discover and select the most appropriate patient and clinical features for the topic of predicting discharge disposition in acute neurological care based on data that are available within 24 h of hospital admission. For instance, discharge weight, ICU and hospital length of stay, days on ventilator, etc. were all promising features that we elected to drop since they would be unavailable at or shortly after admission.

The final dataset for this study was created by selecting and merging 5 such tables (titled *apacheApsVar*, *apachePatientResult*, *apachePredVar*, *patient*, and *admissiondx* [17]) based on the unique patient ID. These five tables were selected after combing through the database and determining that they contained the relevant early admission variables that the study required. To select the patients who only underwent treatment for acute neurological care, we dropped all records except those admitted with a stroke/CVA diagnosis. In the original dataset, there were several discharge categories such as ‘home’, ‘other’, ‘other external’, ‘other hospital’, ‘skilled nursing’, ‘rehab’, ‘death’, and ‘nursing home’. As the goal is to use the predictions as an early alert for healthcare providers, this study focuses on predicting the four typical discharge dispositions. For this purpose, the patients with discharge categories such as ‘other’, ‘other external’, and ‘other hospital’ were discarded from the analysis. ‘Skilled nursing’ and ‘nursing home’ were merged under the ‘nursing facility’ discharge category. As a result, the final four discharge classes were ‘home’, ‘nursing facility’, ‘rehab’, and ‘death’.

As part of the data cleaning, rows with missing discharge outcomes were dropped. Columns missing a relatively small amount of data had their data imputed by taking the median and modes of the data where applicable. For example, missing values in ‘Ethnicity’ column were replaced with the mode whereas missing values in ‘Height’ and ‘Weight’ columns were replaced with the median. A new data column was created extracting the admission hour from the unit admission time. The target outcome discharge locations were extracted and encoded via Python Scikit Learn’s LabelEncoder [19], and all categorical feature columns were encoded using OneHotEncoder [20]. The final dataset contained information from 34 selected feature descriptions of 5,245 acute neurological patients along with their discharge locations.

To perform the binary class (‘home’ vs. ‘non-home’) experiment that this study additionally carries out, the above dataset is further preprocessed to have two discharge outcomes such as ‘home’ vs. ‘non-home’ by combining all patients with ‘nursing facility’, ‘rehab’, and ‘death’ categories to a single ‘non-home’ category.

Table 2 shows a detailed breakdown of these features and their distributions based on the four target discharge outcomes (in case of multi-class outcome experiment) and two target discharge locations (in case of binary-class outcome experiment) along with their statistical significances represented as *p*-values. Table 2 shows that while the binary outcome data is mostly balanced with ‘home’ and ‘non-home’ having roughly equal amount of observations, the multi-class outcome data breakdowns on the other hand exhibits imbalance with ‘home’ being the discharge locations for majority observations. The *p*-values in Table 2 for the discrete categorical features were generated with the chi-squared test, and the *p*-values for the continuous features were generated via the one-way ANOVA test. There are several abbreviations used in Table 2 and they are defined below:

- GCS – Glasgow Coma Score
- MI – Myocardia Infarction
- ICU – Intensive Care Unit
- SICU – Surgical ICU
- MICU – Medical ICU
- CTICU – Cardio-Thoracic ICU
- CSICU – Cardio-Surgical ICU
- CCU – Critical Care Unit
- LOS – Length of Stay

**Table 2:** Breakdown of data per binary-class and multi-class discharge outcomes

**Feature**	**Missing**	**Binary-Outcome data breakdown**			**Multi-outcome data breakdown**				
		**Home**	**Non-Home**	***P*-value**	**Home**	**Nursing**	**Rehab**	**Death**	***P*-Value**
N (%)	-	2612 (49.8%)	2633 (50.2%)	-	2612 (49.8%)	1125 (21.5%)	890 (16.7%)	618 (11.8%)	-
**Patient Demographics**	-	-	-	-	-	-	-	-	-
*Gender*	0	-	-	0.009	-	-	-	-	< 0.001
Female	-	1213 (46.4%)	1328 (50.44%)	-	1213 (46.4%)	647 (57.5%)	376 (42.3%)	305 (49.4%)	-
Male	-	1398 (53.5%)	1305 (49.56%)	-	1398 (53.5%)	478 (42.5%)	514 (57.7%)	313 (50.6%)	-
Age (median) [Q1,Q3]	0	65 [54, 76]	73 [63, 83]	< 0.001	65 [54, 76]	76 [66, 85]	68.5 [58, 77]	75 [64, 83]	< 0.001
*Ethnicity*	33	-	-	< 0.001	-	-	-	-	< 0.001
Caucasian	-	1923 (73.6%)	2111 (80.17%)	-	1923 (73.6%)	903 (80.3%)	721 (81%)	487 (78.8%)	-
African American	-	302 (11.6%)	248 (9.42%)	-	302 (11.6%)	113 (10%)	79 (8.9%)	56 (9%)	-
Hispanic	-	180 (6.89%)	72 (2.73%)	-	180 (6.89%)	18 (1.6%)	19 (2.13%)	35 (5.66%)	-
Native American	-	15 (0.57%)	11 (0.42%)	-	15 (0.57%)	5 (0.44%)	12 (1.35%)	0 (0%)	
Asian	-	52 (1.99%)	46 (1.75%)	-	52 (1.99%)	25 (2.22%)	6 (0.67%)	9 (1.46%)	-
Other/Unknown	-	140 (5.36%)	145 (5.51%)	-	140 (5.36%)	61 (5.42%)	53 (5.96%)	31 (5.02%)	-
**Admission Variables**	-	-	-	-	-	-	-	-	-
*Unit Type*	0	-	-	< 0.001	-	-	-	-	< 0.001
Med-Surg ICU	-	1213 (46.4%)	1216 (46.2%)	-	1213 (46.4%)	529 (47%)	398 (44.7%)	289 (46.8%)	-
Neuro ICU	-	895 (34.3%)	871 (33.08%)	-	895 (34.3%)	390 (34.7%)	279 (31.3%)	202 (32.7%)	-
MICU	-	133 (5.09%)	168 (6.38%)	-	133 (5.09%)	70 (6.22%)	67 (7.53%)	31 (5.02%)	-
SICU	-	131 (5.02%)	207 (7.86%)	-	131 (5.02%)	69 (6.13%	92 (10.3%)	46 (7.44%)	-
Cardiac ICU	-	118 (4.52%)	92 (3.49%)	-	118 (4.52%)	34 (3.02%)	35 (3.93%)	23 (3.72%)	-
CCU-CTICU	-	69 (2.64%)	52 (1.97%)	-	69 (2.64%)	22 (1.96%)	14 (1.57%)	16 (2.59%)	-
CTICU	-	44 (1.69%)	15 (0.57%)	-	44 (1.69%)	5 (0.44%)	1 (0.11%)	9 (1.46%)	-
CSICU	-	9 (0.35%)	12 (0.46%)	-	9 (0.35%)	6 (0.53%)	4 (0.45%)	2 (0.32%)	-
*Unit Stay Type*	0	-	-	0.163	-	-	-	-	0.031
Admit	-	2434 (93.2%)	2436 (92.52%)	-	2434 (93.2%)	1051 (93.4%)	818 (91.9%)	567 (91.7%)	-
Transfer	-	110 (4.21%)	105 (3.99%	-	110 (4.21%)	30 (2.67%)	42 (4.72%)	33 (5.34%)	-
Readmit	-	68 (2.60%)	92 (3.49%)	-	68 (2.60%)	44 (3.91%)	30 (3.37%)	18 (2.91%)	-
Admission Hour (mean)[SD]	0	11.27 [8.08]	11.27 [8.08]	0.443	11.27 [8.08]	11.15 [8.15]	11.76 [8.15]	11.51 [7.64]	0.324
*Comorbidities*	-	-	-	-	-	-	-	-	-
Leukemia	0	10 (0.38%)	13 (0.49%)	0.69	10 (0.38%)	5 (0.44%)	4 (0.45%)	4 (0.65%)	0.848
AIDS	0	1 (0.04%)	0 (0%)	0.997	1 (0.04%)	0 (0%)	0 (0%)	0 (0%)	0.799
Hepatic Failure	0	11 (0.42%)	12 (0.46%)	0.985	11 (0.42%)	3 (0.27%)	4 (0.45%)	5 (0.81%)	0.436
Cirrhosis	0	11 (0.42%)	15 (0.57%)	0.569	11 (0.42%)	6 (0.53%)	3 (0.34%)	6 (0.97%)	0.307
Immunosuppression	0	30 (1.14%)	28 (1.06%)	0.871	30 (1.14%)	9 (0.80%)	10 (1.12%)	9 (1.46%)	0.636
Lymphoma	0	9 (0.35%)	10 (0.38%)	0.986	9 (0.35%)	2 (0.18%)	6 (0.67%)	2 (0.32%)	0.32
Metastatic Cancer	0	22 (0.84%)	26 (0.99%)	0.684	22 (0.84%)	9 (0.80%)	7 (0.79%)	10 (1.62%)	0.278
Diabetes	0	527 (20.2%)	556 (21.12%)	0.42	527 (20.2%)	251 (22.3%)	180 (20.2%)	125 (20.2%)	0.49
MI (Last 6 months)	0	11 (0.42%)	14 (0.53%)	0.703	11 (0.42%)	6 (0.53%)	4 (0.45%)	4 (0.65%)	0.887
**Clinical Information**	-	-	-	-	-	-	-	-	-
Height (median) [Q1,Q3]	124	169.4 [162,178]	167.94 [160, 178]	< 0.001	169.4 [162,178]	166.36 [160,175]	169.85 [163,178]	168.08 [160,178]	< 0.001
Weight (median,kg) [Q1,Q3]	213	87.44 [69.4, 99]	84.66 [66, 97]	< 0.001	87.44 [69.4, 99]	81.70 [63,93]	88.58 [71,101]	84.4 [67,95.6]	< 0.001
Temperature (mean, C) [SD]	0	36.49 [0.54]	36.50 [0.71]	0.292	36.49 [0.54]	36.50 [0.54]	36.53 [0.62]	36.47 [1.01]	0.197
Respiratory Rate (mean) [SD]	0	23.67 [15.1]	26.34 [14.98]	< 0.001	23.67 [15.1]	26.1 [14.49]	26.05 [15.35]	27.23 [15.30]	< 0.001
Intubated	0	162 (6.20%)	611 (23.21%)	< 0.001	162 (6.20%)	160 (14.2%)	104 (11.7%)	347 (56.1%)	< 0.001
Ventilator	0	184 (7.04%)	651 (24.72%)	< 0.001	184 (7.04%)	181 (16.1%)	113 (12.7%)	357 (57.8%)	< 0.001
Mean BP (mean) [SD]	0	104.70 [38.56]	111.22 [41.92]	< 0.001	104.70 [38.56]	111.68 [41.12]	114.5 [39.36]	105.66 [46.24]	< 0.001
Heartrate (mean) [SD]	0	83.99 [29.21]	95.39 [31.06]	< 0.001	83.99 [29.21]	93.79 [29.85]	91.02 [28.99]	104.58 [34.13]	< 0.001
Dialysis	0	26 (0.99%)	50 (1.90%)	0.008	26 (0.99%)	25 (2.22%)	10 (1.12%)	15 (2.43%)	0.004
Glucose (mean) [SD]	0	129.23 [63.53]	146.67 [74,84]	< 0.001	129.23 [63.53]	138.11 [67.30]	142.82 [74.96]	167.77 [83.31]	< 0.001
GCSEyes (mean) [SD]	0	3.71 [0.66]	3.13 [1.11]	< 0.001	3.71 [0.66]	3.3 [0.97]	3.48 [0.83]	2.32 [1.31]	< 0.001
GCSVerbal (mean) [SD]	0	4.32 [1.25]	3.26 [1.69]	< 0.001	4.32 [1.25]	3.4 [1.60]	3.81 [1.52]	2.21 [1.62]	< 0.001
GCSMotor (mean) [SD]	0	5.77 [0.78]	5.15 [1.46]	< 0.001	5.77 [0.78]	5.42 [1.09]	5.61 [0.96]	3.99 [1.95]	< 0.001
**Apache Variables**	-	-	-	-	-	-	-	-	-
Apache Score (mean) [SD]	727	42.11 [15.5]	55.76 [21.92]	< 0.001	42.11 [15.5]	53.68 [17.87]	46.97 [17.23]	72.19 [25.51]	< 0.001
Acute Physiology Score(mean)[SD]	727	30.13 [12.87]	40.79 [20.88]	< 0.001	30.13 [12.87]	37.61 [16.52]	33.86 [15.55]	56.56 [26.01]	< 0.001
Predicted ICU Mortality (mean) [SD]	727	0.05 [0.06]	0.11 [0.15]	< 0.001	0.05 [0.06]	0.09 [0.11]	0.06 [0.09]	0.23 [0.20]	< 0.001
Predicted Hospital Mortality (mean) [SD]	727	0.11 [0.10]	0.21 [0.19]	< 0.001	0.11 [0.10]	0.19 [0.15]	0.14 [0.13]	0.36 [0.24]	< 0.001
Predicted ICU LOS (mean) [SD]	727	2.64 [1.02]	3.44[1.70]	< 0.001	2.64 [1.02]	3.14 [1.45]	2.97 [1.32]	4.66 [2.01]	< 0.001
Predicted Hospital LOS (mean) [SD]	727	8.13 [2.20]	9.19 [2.90]	< 0.001	8.13 [2.20]	8.96 [2.91]	8.63 [2.53]	10.44 [3.01]	< 0.001

## Methodology

Jupyter Notebooks [21] and Google Cloud Platform [22] were the primary development environments. Python 3 [23] was used as the development language with Scikit Learn [24] as the primary library for the ML aspects. For both experiments, a range of classification algorithms were utilized and compared to build the models and identify the best performer. The five algorithms [25] selected were Random Forest (RF), XGBoost (XGBC), Support Vector Machine (SVM), K-Nearest Neighbor (KNN), and Logistic Regression (LR). These models were primarily chosen based upon the occurrence frequency and performance observed from the background literature. For instance, [16] uses KNN, [8, 13, 16] employed RF and LR, [8, 10, 12, 16] employed XGB, and [10, 12] utilized SVM.

For this article, the performance of the classifiers is evaluated using various standard evaluation metrics, namely precision, recall, specificity, F1 score, and area under the receiver operating characteristic (ROC) curve. The curated dataset was divided into training (75%) and testing (25%) sets, and those sets were utilized during model training and prediction evaluation respectively. During the data split, stratified sampling was enforced to ensure that a reasonable number of instances were sampled from each discharge category to guarantee that the test set was representative of the overall population. The five algorithms were optimized via hyperparameter tuning to find the best-performing parameter distribution. The optimization approach was the same for both experiments, using a combination of *RandomizedSearchCV* [25]*, GridSearchCV* [25]*,* as well as other iterative approaches for determining the best parameter distribution depending on the algorithm being optimized. *RandomizedSearchCV* takes a dictionary of parameter distributions and tries out a number of cross-validated parameter settings that are sampled from those specified parameter distributions, while *GridSearchCV* exhausts all possible parameter combinations. While the grid search can return a higher-quality solution, the runtime can grow exponentially with a large number of parameters to test i.e. in the cases of the tree-based algorithms.

For the tree-based algorithms with a high number of parameters i.e. RF and XGBoost, the randomized search with 1,000 sampled parameter combinations and fivefold cross validation was used totaling 5,000 fits to sample a range of specified parameters. For the SVM, the fivefold cross-validated grid search was used due to SVM having a much lower number of possible parameters, totaling 500 fits. For KNN, a range of K from 1 to 50 to find the optimal K-value was sampled. With LR, an iterative approach trying each solver over a range of values for the regularization parameter was taken. All optimization was targeted to improve the F1 score metric accuracy.

For exploring interpretability of the best-performing model, an explainable AI framework known as SHAP (Shapley Additive exPlanations) was utilized. SHAP is an approach to explaining output from ML models using game theory, or specifically *Shapley values* [26]. In this case, a feature value is assumed to be a ‘player’ in a game where the ‘payout’ of that game is the prediction [27]. *Shapley values* is a method from coalitional game theory that tells us how to fairly distribute that ‘payout’, i.e. importance, among the features used [27]. We used the SHAP *summary_plot* method on the best-performing model. *Summary_plot* combines the feature importance and feature effects, with each point on the plot being a Shapley value for a feature and an instance with the features being ordered by importance. The position on the y-axis is determined by the feature and the position on the x-axis determined by the Shapley value [27]. Features on the plot are ordered by their importance with the color representing the feature value from low to high providing an indication of the relationship between a feature’s value and its impact on the prediction [27].

## Results and discussion

In this section, first the results achieved from the multi-class model are presented and discussed followed by the results gained from the binary-class model.

### Multi-class experiment results

The results for the best-performing hyperparameter optimization of the multi-class outcome models as achieved by the methodology outlined in "Methodology" section are as follows:

- LR – solver = L-BFGS, C = 0.01, penalty = l2
- SVM – C = 0.001, gamma = 0.001, kernel = linear
- KNN – K = 8, metric = Minkowski Distance
- XG – max_depth = 8, n_estimators = 747
- RF – max_depth = 10, min_samples_split = 10, n_estimators – 700

Table 3 below gives the results of the multi-class models on the test dataset with the four outcome classes as rows and the metrics and models as columns and subcolumns respectively. The most important observation is that the top-performing models (XGBC and RF) performed relatively well across all four metrics in distinguishing the correct outcome classes. KNN proves to be the least effective model due to its relatively poor scoring across the board of evaluation metrics.

**Table 3:** Evaluation of the multi-class model with test data

	**Precision**					**Recall**				
	LR	SVM	KNN	XG	RF	LR	SVM	KNN	XG	RF
Home	71%	55%	71%	64%	66%	53%	63%	36%	61%	68%
Death	44%	45%	52%	48%	49%	57%	55%	54%	56%	54%
rehab	23%	27%	42%	27%	36%	30%	27%	38%	31%	46%
Nursng	36%	38%	40%	36%	33%	44%	42%	32%	43%	22%
	**F1**					**AUC**				
	LR	SVM	KNN	XG	RF	LR	SVM	KNN	XG	RF
Home	58%	60%	44%	69%	70%	85%	78%	81%	85%	86%
Death	47%	27%	44%	44%	47%	74%	63%	71%	73%	77%
rehab	29%	20%	27%	21%	23%	61%	57%	56%	60%	61%
Nursng	35%	27%	34%	35%	37%	66%	61%	61%	66%	68%

**Fig. 1:**
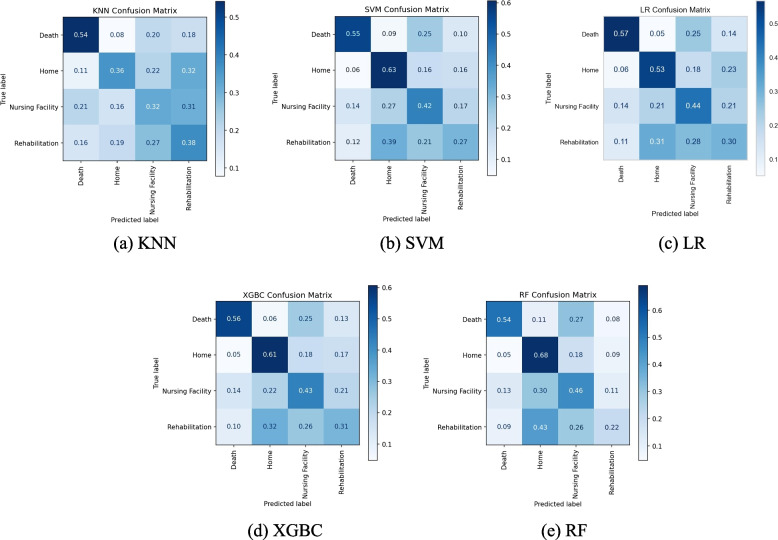
Confusion Matrices of All 5 Models Based on Multi-class Test Dataset

Confusion matrices are a great tool in gaining further insight into these types of multi-classification problems and are shown in Fig. 1(a-e). Based on the test dataset, the KNN model (Fig. 1.a) predicts the class ‘death’ with a 54% recall (i.e., 54% of the time ‘death’ observations are classified correctly as ‘death’ by the model), with the remaining three classes all having similar recall percentages in the range of 32%-38%. Figure 1(a) also depicts that the primary errors in the KNN model come from the classes of ‘home’, ‘nursing facility’, and ‘rehab’. Actual ‘home’ classes are being incorrectly predicted as ‘nursing facility’ and ‘rehab’ 22% and 32% of the time respectively. Similarly, ‘nursing facility’ is incorrectly predicted as ‘rehab’ 31% of the time, and ‘rehab’ incorrectly as ‘nursing facility’ 27% of the time which significantly impacts the overall accuracy. These results suggest that the KNN is not effective enough in characterizing and distinguishing these three discharge classes.

Figure 1.b and c show the confusion matrices for SVM and LR, which are somewhat similar to the KNN matrix. For SVM, the ‘home’ class is the most accurately predicted one with a 63% recall, while for the LR model the ‘death’ class is at the top with 57% recall. The SVM model tends to erroneously predict ‘death’ as ‘nursing facility’ 25% of the time, similar to the LR model. Figure 1.d and e give the matrices for the tree-based models, XGBC and RF. These models have very similar prediction distributions with both ‘home’ and ‘death’ classes being predicted with relatively better accuracies. However, they both tend to heavily predict the actual labels of ‘nursing facility’ and ‘rehab’ incorrectly as ‘home’ between 41–52% of the time. This could be an indicator that these models are overfitting the training data with the majority ‘home’ class.

Furthermore, as evidenced by these confusion matrices, nearly all models struggled with predicting accurately between ‘home’, ‘nursing facility’, and ‘rehab’. This could likely be remedied by having more insightful patient features, such as information regarding their physical therapy sessions while in the care unit or the patient’s Activity Measure for Post-Acute Care (AM-PAC) [28] scores that are commonly used in this kind of medical setting to give the models the necessary ability to distinguish between these three relatively similar discharge outcomes. Additionally, features relating to the patient’s insurance information, the patient’s home/family situation and expected assistance would also assist the models in distinguishing between these three outcomes. However, there were no such features available in the utilized dataset.

**Fig. 2:**
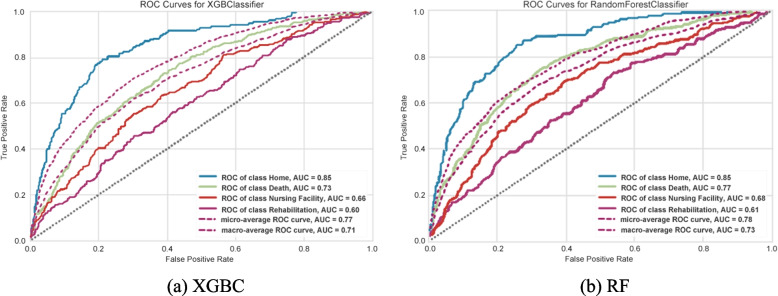
ROC Curves of the XGBC and RF Model Based on Multi-class Test Dataset

ROC curves were created by binarizing the output on a per-class basis resulting in a curve for each class, per model, for the best-performing models (XGBC and RF) as represented by Fig. 2 (a, b). The ROC curves indicate that the ‘home’ class has a much larger accuracy (AUC value or c-statistic), showing that both models had an easier time correctly distinguishing the ‘home’ class from other classes. Also, both models seemed to have a respectable AUC values when considering the number of outcome classes. With this graph format, the micro and macro averages are also given, where the micro average is a weighted average of the curves based on class balance and the macro average is the true average of the four curves. There is an occurring trend where the ‘home’ curve performs well above the averages, the death curve performs in between or slightly above the averages, and ‘rehab’ and ‘nursing facility’ performing under the averages. This reinforces that the models lack the information and ability to consistently distinguish the ‘nursing facility’ and ‘rehab’ classes.

**Fig. 3:**
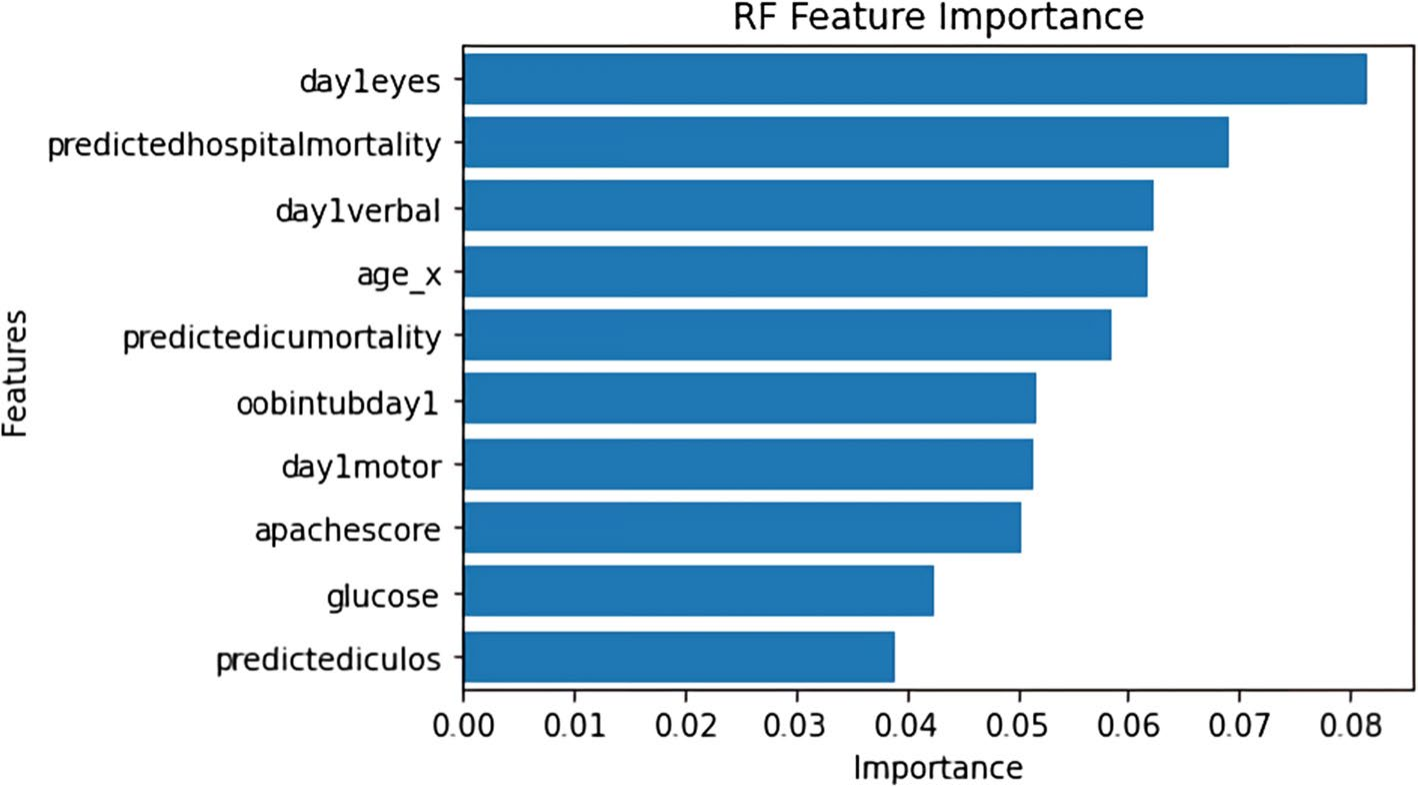
Multi-class Feature Importance Graph of the RF Model

**Fig. 4:**
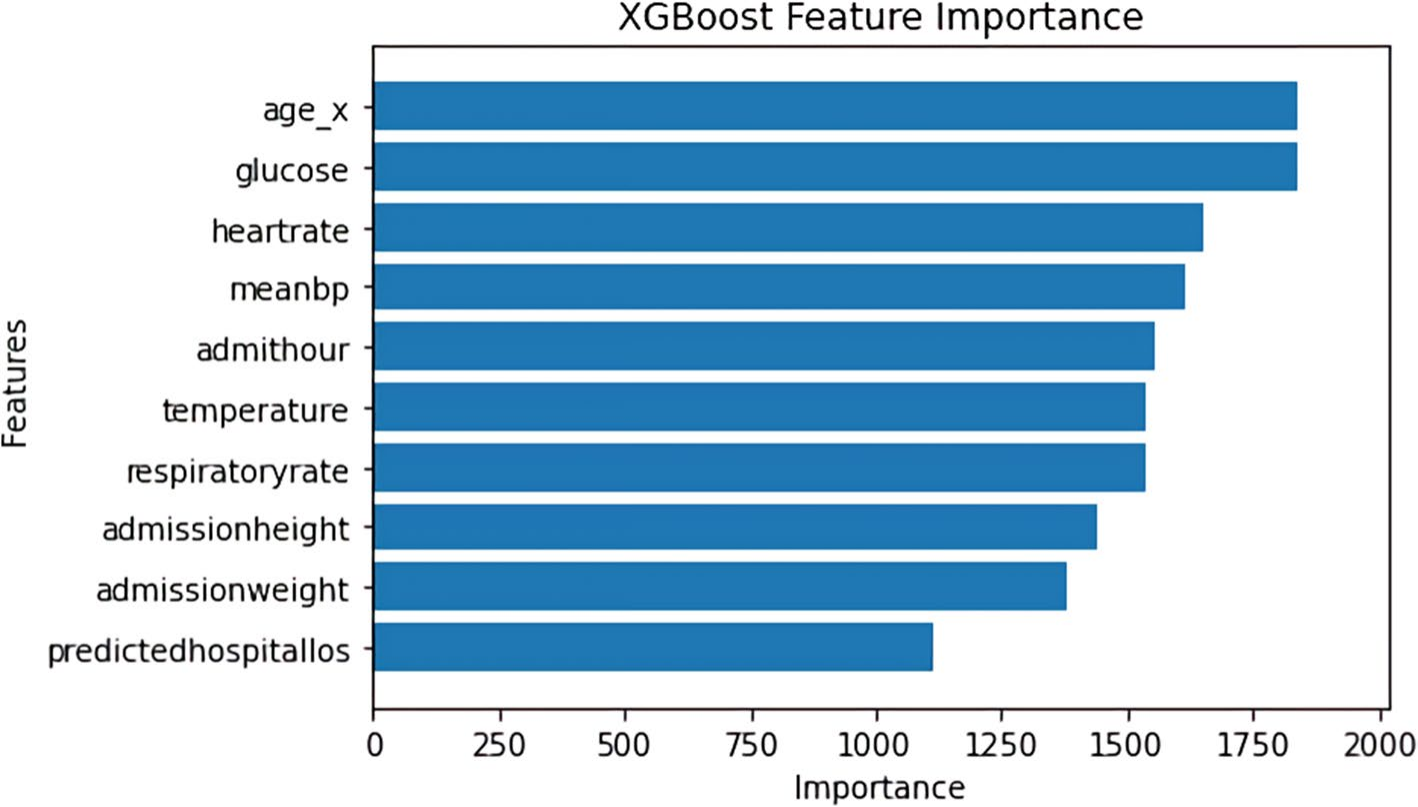
Multi-class Feature Importance Graph of the XGBoost Model

Figures 3 and 4 below show the feature importance for the best-performing RF and XGBC models respectively. Figure 3 uses the *permutation_importance* [24] method of Scikit-Learn to calculate the importance of each feature in the RF model’s decision. This method uses an algorithm to randomly shuffle features values and check its effect on the model accuracy score, while the XGBoost method *plot_importance* [24] using the ‘weight’ importance type*,* plots the number of times the model splits its decision tree on a feature as depicted in Fig. 4. The important features that are common to the both graphs are age and glucose. The RF model found hospital, ICU mortality predictions, and the GCS scores (i.e. eye, verbal, motor) as the significant features in predicting the discharge locations, whereas the XGBC model preferred mean blood pressure, heartrate, and other admission features such as age, height, and weight.

**Fig. 5:**
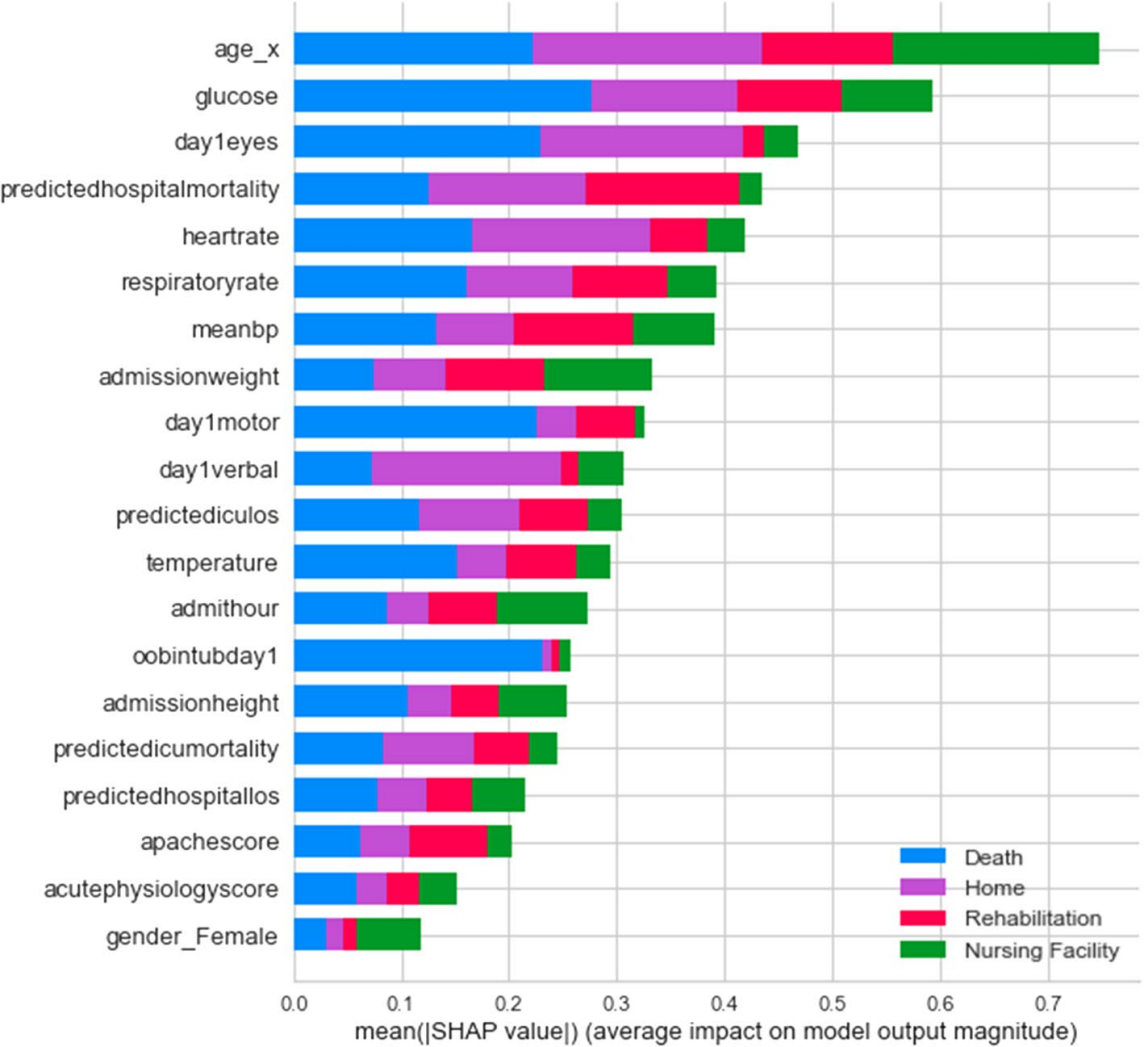
SHAP Summary Plot Showing Feature Value Impact on XGBoost Multi-class Model Outputs

Figure 5 shows the SHAP summary plot for the multi-class experiment with the best-performing XGBC model. This gives us additional insights and provides a breakdown of feature importance based around the feature’s impact on each target class as indicated by the different colors of the bar. The results of this graph somewhat coincide with the graph of feature importance of the XGBC model in Fig. 4, as age, glucose, and heartrate are all in the top five most important features in both graphs. The patient’s age has the highest impact on the ‘home’ and ‘death’ outcome categories and the patient’s glucose readings also have a large impact on the ‘death’ class. Another interesting observation is that the patient’s day one GCS motor score and whether they were intubated have an extremely high impact correlation to the ‘death’ category. Furthermore, the GCS verbal scores indicate they have a high impact on the ‘home’ category. Similarly, average blood pressure (meanBP) seems to drive ‘rehab’ and admission weight seems to impact ‘nursing facility’ categories.

### Binary-class experiment results

The results for the best-performing hyperparameter optimization of the binary outcome models as achieved by the methodolody outlined in "Methodology" section are as follows:

- LR – solver = L-BFGS, C = 0.1, penalty = l2
- SVM – C = 0.1, gamma = 0.001, kernel = linear
- KNN – K = 9, metric = Minkowski Distance
- XG – max_depth = 5, n_estimators = 285
- RF – max_depth = 100, min_samples_split = 10, n_estimators = 100

The results of the binary experiment on the test dataset is shown below in Table 4. As expected, all models exhibited considerable improvements in accuracy while predicting ‘home’ vs. ‘non-home’ classes compared to accuracies that were achieved during multi-class prediction. The binary class model performs similarly to the multi-class model at 70% F1 value when predicting ‘home’ class using XGBC. However, unlike the multi-class, the binary model’s performance improved significantly when the prediction was made for the ‘non-home’ class at 70% F1 score. Like the multi-class experiment, the top-performing models are once again XGBC and RF considering both ‘home’ and ‘non-home’ accuracies. Contrary to the multi-class however, the KNN model had a more solid performance with an AUC of 72% indicating less confusion between the two outcome classes as compared to the four classes.

**Table 4:** Evaluation of the binary class model with binary test data

	**Precision**					**Recall**				
	LR	SVM	KNN	XG	RF	LR	SVM	KNN	XG	RF
Home	68%	67%	64%	70%	69%	75%	76%	78%	71%	72%
Non-Home	72%	72%	72%	71%	71%	65%	63%	56%	70%	69%
	**F1**					**AUC**				
	LR	SVM	KNN	XG	RF	LR	SVM	KNN	XG	RF
Home	71%	71%	70%	70%	70%	76%	76%	72%	77%	76%
Non-Home	68%	67%	63%	70%	70%					

**Fig. 6:**
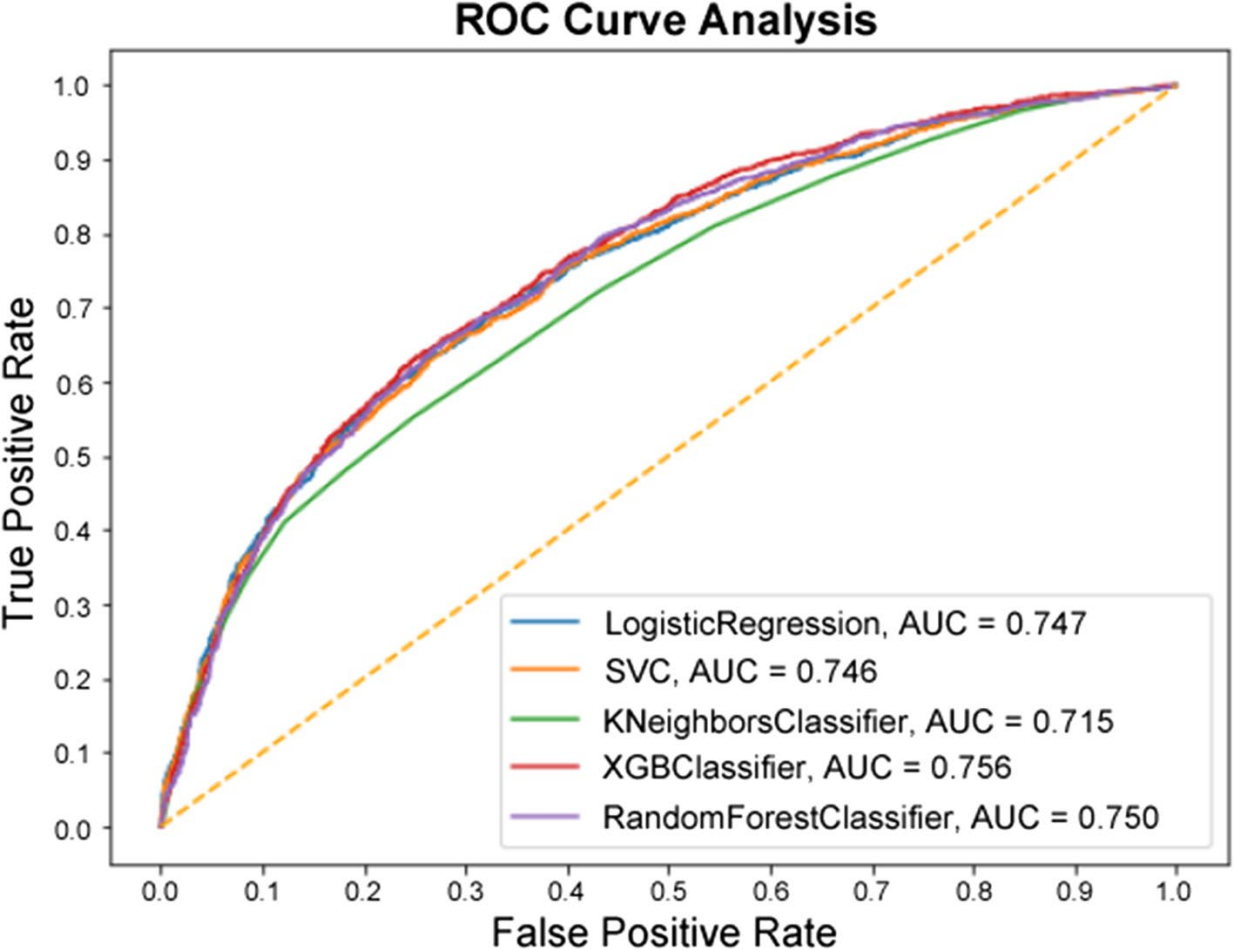
ROC Curve of the Binary Experiment

Since in the binary experiment there are only two classes, the ROC curve (Fig. 6) is able to be constructed as it normally would. The top four models all performed very similarly in the ROC analysis, all being within a percentage point of one another. The KNN model being the exception, with an AUC of 71.5% compared to the best-performing XGBC at 75.6%.

**Fig. 7:**
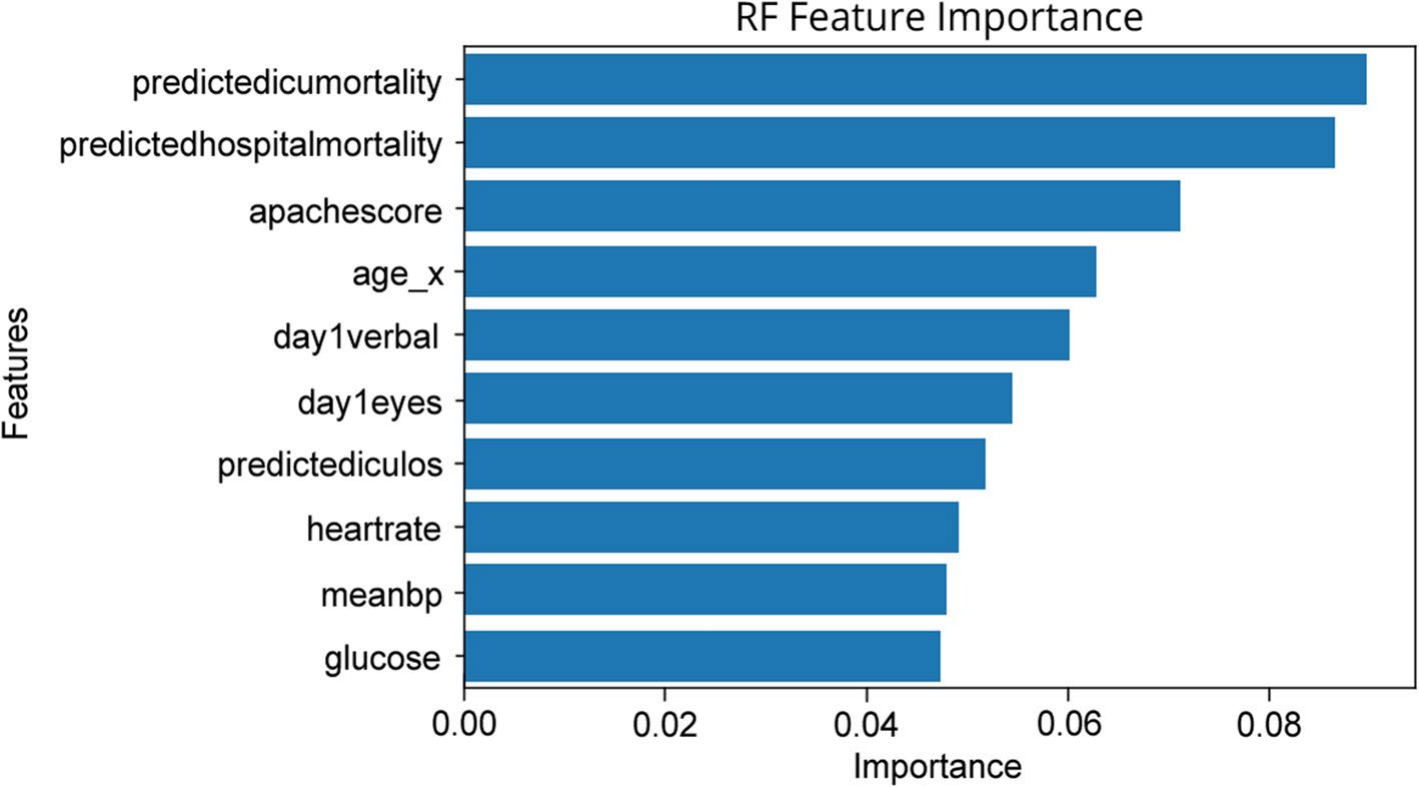
Binary Feature Importance Graph of the RF Model

**Fig. 8:**
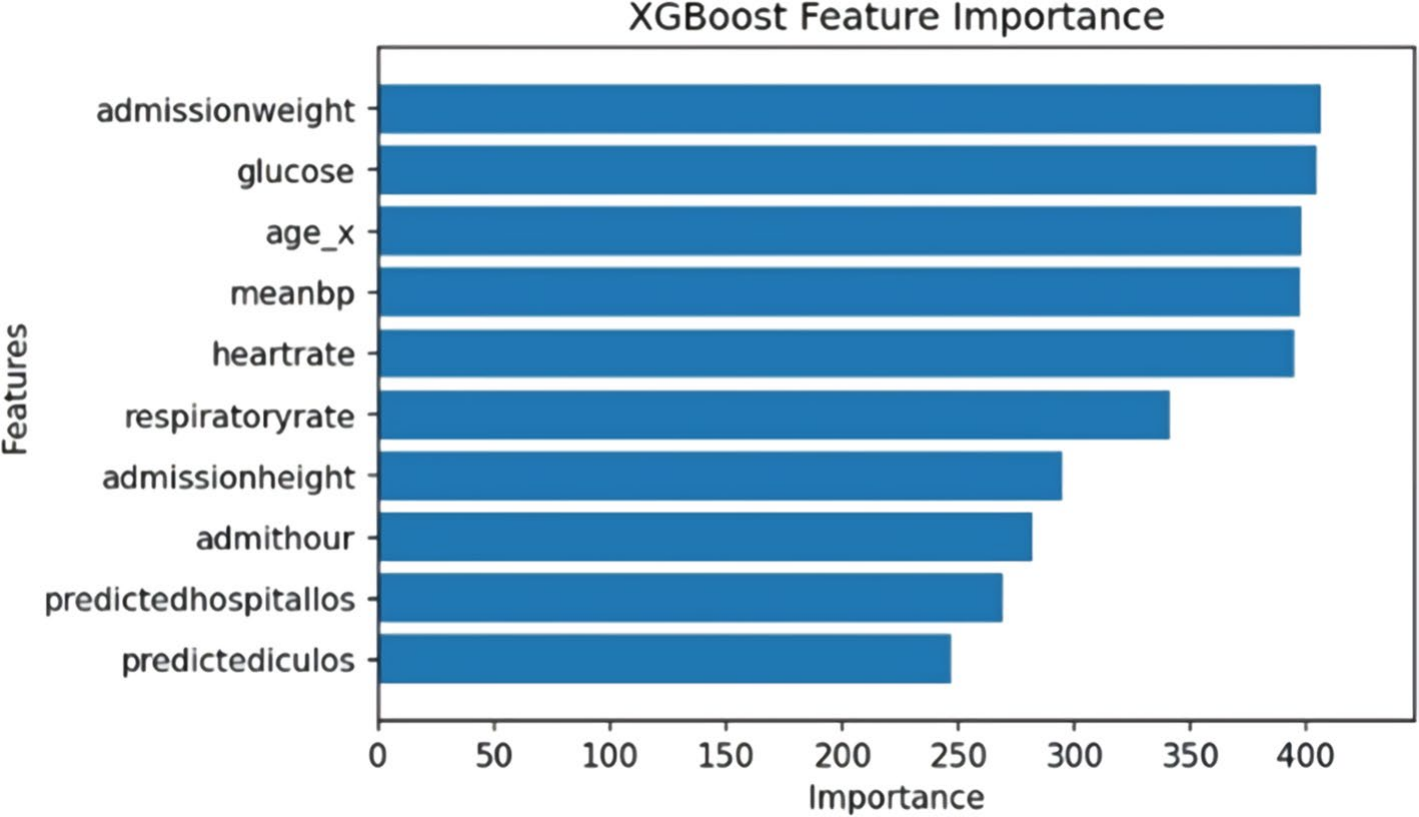
Binary Feature Importance Graph of the XGBoost Model

The feature importance graphs for RF and XGBC (Figs. 7 and 8) for the binary experiment are noticably similar to the two from the multi-class experiment. For RF, the top features are shuffled a bit, and the intubation day 1 and GCS motor score are replaced by heartrate and mean blood pressure. For XGBC, temperature is replaced by predicted ICU LOS, with the order shuffled to have admission weight at the top slightly edging out glucose and age.

**Fig. 9:**
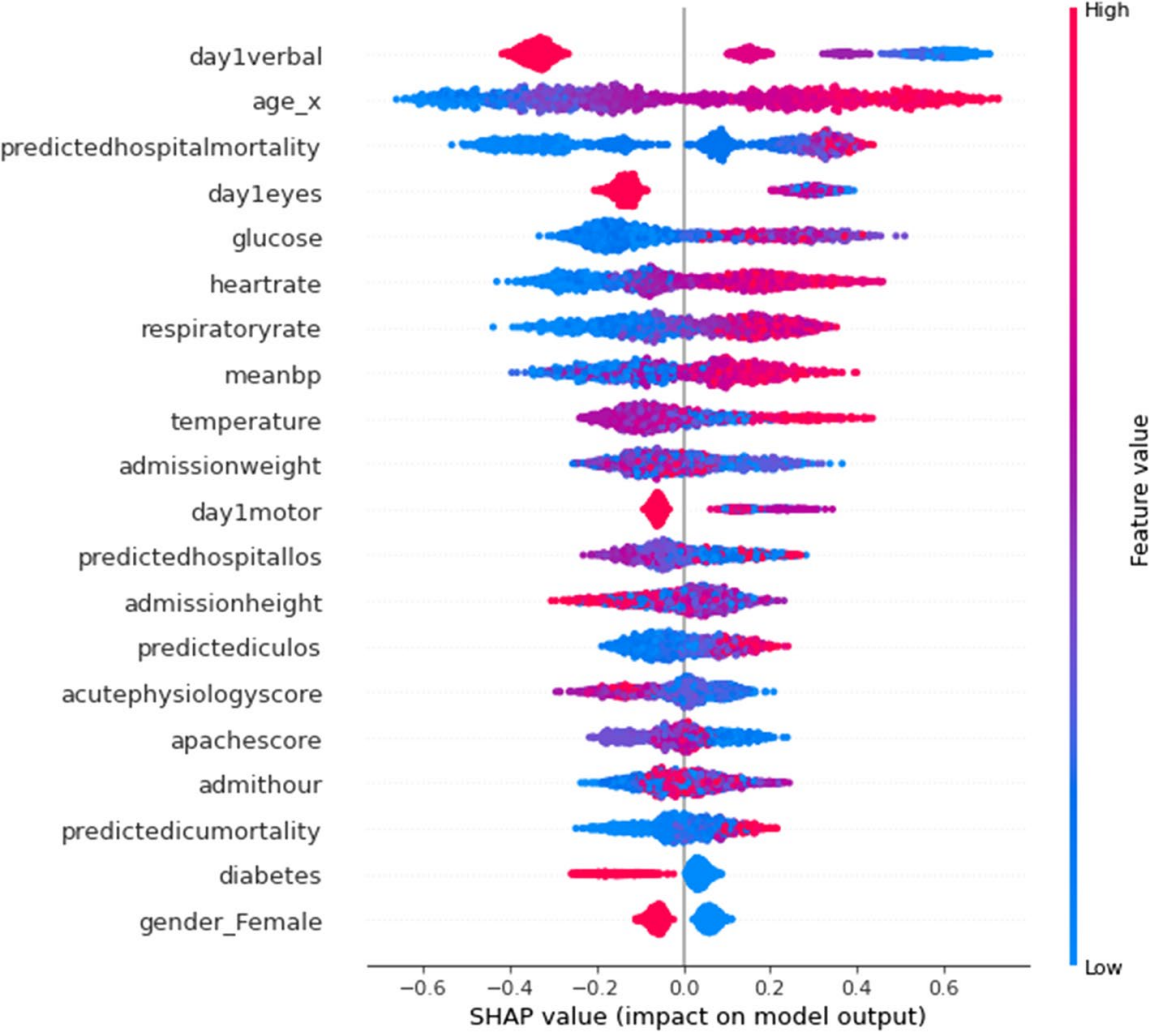
SHAP Summary Plot for XGBoost Binary-class Model

Figure 9 displays a SHAP summary plot for the features of the XGBC model. This graph indicates the top features ranked by importance and shows how feature values impact the model’s output with the colors indicating higher feature values for red, and lower values for blue, with overlapping values jittered on the y-axis. The x-axis is the SHAP value which indicates the impact on the model’s output, in this case with positive values trending toward the ‘non-home’ class, and negative trending toward the ‘home’ class.

Some takeaways from this graph are that when a patient scores higher values on the GCS for eye and verbal response, the more likely they are to be discharged to ‘home’, according to the model. This conclusion is reflected in the plot by having most higher (red) GCS eye and verbal features values concentrating on the ‘home’ side of the classification. Another distinctive conclusion is that the higher the patient’s age, the more likely it is for a ‘non-home’ discharge. Similarly, lower values for glucose, meanBP, and heartrates indicate a ‘home’ discharge. However, it should be noted that these effects simply describe the model’s behavior and are not necessarily indicative of a causal relationship in the real world [27].

### Translating Study Results to a Clinical Care Setting

**Fig. 10:**
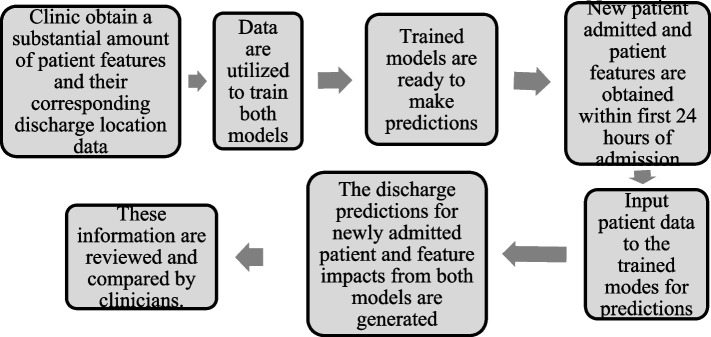
Overall process within the context of a clinic

This section aims to clarify the results of this article and discuss its practical implications regarding how these models and results could be used in a clinical context. Figure 10 shows the overall process within the context of a healthcare clinic. In most cases, one of the immediate concerns of clinicians is to know whether a patient will likely be discharged to their home, or other locations which typically require advanced planning and arrangements with entities such as insurers, hospitals, and the patients/patient families. For this context, this study developed a simple binary-class (‘home’, vs. ‘non-home’) model that predicts the most likely discharge destination for CVA patients using variables and patient characteristics obtained within the first 24 h of hospital stay. The study further developed another model that is able to generate early predictions while distinguishing between four discharge outcomes (‘home’, ‘death’, ‘rehab’, ‘nursing facility’) in cases where more insight is needed for the potential discharge destinations. Our study results show that, once trained with substantial amount of labeled discharge disposition and patient data, both models are able to generate predictions after 24 h of a patient’s hospital stay with acceptable accuracies. These models and the predictions could therefore be used by clinicians both in a suggestive context (prior to the clinicians making their determination on patient discharge location) or a confirmative context (after the clinicians have made their determinations) to aid healthcare providers in planning effective, timely, and equitable discharge recommendations. There is further advantage of having two parallel models generating predictions simultaneously based on the same input features. For example, if there is any disagreement between the models (i.e., the binary model disagreeing with the multi-class outcome models, or the model predictions agreeing with each other, yet disagreeing with the clinician’s determination), then clinicians could consider the patient’s case to be reevaluated more closely.

**Fig. 11:**
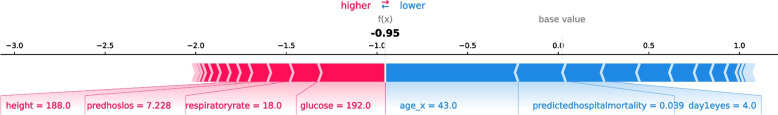
SHAP force plot showing the values and directions of features causing a singular instance correctly predicted as ‘Home’

**Fig. 12:**
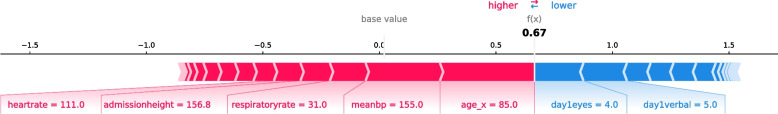
SHAP force plot showing the values and directions of features causing a singular instance correctly predicted as ‘Non-Home’

This study also shows that instead of having a black box model generating predictions, it is possible to augment the predictions with visual and interpretable representations of patient characteristics driving these predictions which will likely to make the results more trustworthy and explainable to the clinicians. Feature importance plots (Figs. 3, 4, 7, and 8) and SHAP summary plots (Figs. 5 and 9) can be integrated as part of the prediction application tool and potentially can provide important and interpretable insights about the predictions. Additionally, clinicians can further investigate a particular patient’s data and the corresponding prediction more closely by utilizing SHAP’s force plots [27, 28] as shown in Figs. 11 and 12. These plots allow for a visualization of the most impactful features on a single patient record instance from the data, allowing for more transparency and interpretability on a case-by-case basis. Force plots show the prediction value (bolded number) yielded by the SHAP values of each particular instance, which is then transformed to either 0 or 1, depending on the model’s prediction based on the data. The red force values are the value pushing that prediction value closer to 1(non-home), whereas the blue force values are the values pushing the prediction toward 0 (home). The instance where the model correctly predicts ‘home’ (Fig. 11) indicates that the lower values (represented by negative numbers after normalizing the data) of age, predicted hospital mortality, and the meanBP of the patient were among the top 3 features used by the model that resulted in the ‘home’ prediction for this particular patient. In Fig. 12, we see that the higher values of the age, respiratory rate, and the mean blood pressure are the primary features driving the model to correctly predict the discharge outcome of ‘non-home’.

At such a point, clinicians could look at which variables and patient characteristics are driving the model’s prediction to investigate further should they so choose. With this, clinicians achieve a higher level of confidence in their ultimate determination backed by the model’s data-driven predictions and the ultimate goal of providing the highest-quality level of care for the patient could be achieved. By providing this optimal level of care and prediction, common negative consequences such as hospital readmittance/reinjury due to improper discharge location and hospital-acquired infections due to patient overstay, could potentially be avoided.

### Limitations of this study

There are several limitations present in this study that should be noted. As stated previously, the predications of the models are based upon the first 24 h of available data intended to provide a prompt expeditious prediction. Due to the premise of the research focusing on patients who were admitted with CVAs, and the fact that the study focuses only on patient variables that can be acquired within the first day of admission so that physicians may be informed as early as possible, the total amount usable records acquired from the eICU-CRD was not very large. Many of the noticed error patterns with our model predictions could plausibly be remedied simply by having access to more data to feed into them belonging to those underrepresented classes.

Additionally, the models only account for past practice of the hospitals and healthcare providers for the given dates of the dataset which does not necessarily indicate current or optimal practice in discharge decision-making. On top of that, additional factors such as insurance provider input, bed space availability in local facilities, as well as preferences of the patients’ families all can play a big role in the ultimate destinations of the patients. None of these factors are explicitly considered by these models or provided by the database used. Therefore as these factors change, potentially so too would the ultimate patient discharge outcomes.

## Conclusion

In this study, two parallel experiments were conducted that compared the performance of five different ML algorithmic approaches for predicting discharge dispositions based on patient data available within 24 h of hospital admission. Our first experiment built and evaluated models in order to predict four discharge locations such as ‘home’, ‘nursing facility’, ‘rehab’, and ‘death’ and achieved acceptable accuracies when predicting the ‘home’ discharge class while exhibiting difficulty in distinguishing ‘nursing facility’ and ‘rehab’ discharge classes from other outcomes. The results show that the model could be improved by having additional features such as ‘patient mobility’ or ‘past history of physical therapy needs’ added to the dataset that could potentially provide additional insight to firmly characterize patients with ‘nursing facility’ and ‘rehab’ discharge needs. The second experiment focuses on predicting ‘home’ vs. ‘non-home’ discharge locations. It is observed that during the early stage of hospital admission, knowing whether a patient could be discharged to ‘home’ rather than any other destinations may be of significant interest for both the patient and the facility. The results of the second experiment suggest that the best performing model was able to distinguish between ‘home’ vs. ‘non-home’ discharge locations with respectable accuracy. This study used various hyperparameter tuning methods to optimize the models and was able to achieve the best results with the RF and XGBC model in both experiments. The accuracy results achieved from the best model show promising results in predicting acute neurological patient discharge destinations based on a patient’s basic demographic, clinical, and medical risk assessment information acquired within the first 24 h. This study concludes that such models could potentially be used to better inform medical professionals, as well as patient families, to allow them to deliver higher-quality patient care as well as more appropriate discharge planning.

### Future Work

In the future, this research will focus on externally validating these results, as well as implementing deep learning algorithms to create predictive models with the hope of discovering greater accuracies and complex hidden patterns which were not yet explored by the traditional ML algorithms utilized in this study. To be able to effectively implement deep learning, a much larger dataset will be required as the performance and benefits of deep learning generally increase relative to the amount of data available as input.

Furthermore, additional research on how these models perform with the exclusion of the ‘death’ outcome will be undertaken. While expiry is a common outcome with these types of severe neurological diagnoses that also often requires its own advanced planning, there may be some significance in results obtained by excluding it that may allow for clearer interpretation of the ‘non-home’ category for clinicians.

## Data Availability

The datasets analyzed during the current study are available in the eICU-CRD repository, https://eicu-crd.mit.edu/
